# Abundance and Distribution of Sperm Whales in the Canary Islands: Can Sperm Whales in the Archipelago Sustain the Current Level of Ship-Strike Mortalities?

**DOI:** 10.1371/journal.pone.0150660

**Published:** 2016-03-21

**Authors:** Andrea Fais, Tim P. Lewis, Daniel P. Zitterbart, Omar Álvarez, Ana Tejedor, Natacha Aguilar Soto

**Affiliations:** 1 Biodiversidad, Ecología Marina y Conservación (BIOECOMAC), Dept. Animal Biology, Geology and Edaphology, La Laguna University, Tenerife, Canary Islands, Spain; 2 Ocean Acoustics Lab, Alfred-Wegener-Institut Helmholtz-Zentrum für Polar-und Meeresforschung, Bremerhaven, Germany; 3 Applied Ocean Physics and Engineering, Woods Hole Oceanographic Institution, Woods Hole, Massachusetts, United States of America; 4 CIMA Canarias, Avda. Los Majuelos 115, 38107 Santa Cruz de Tenerife, Tenerife, Canary Islands, Spain; 5 KAI Marine Services, Nalon 16, 28240, Hoyo de Manzanares, Madrid, Spain; 6 CREEM, Centre for Research into Ecological and Environmental Modelling, Scottish Oceans Institute, University of St. Andrews, St Andrews, Scotland; Virginia Commonwealth University, UNITED STATES

## Abstract

Sperm whales are present in the Canary Islands year-round, suggesting that the archipelago is an important area for this species in the North Atlantic. However, the area experiences one of the highest reported rates of sperm whale ship-strike in the world. Here we investigate if the number of sperm whales found in the archipelago can sustain the current rate of ship-strike mortality. The results of this study may also have implications for offshore areas where concentrations of sperm whales may coincide with high densities of ship traffic, but where ship-strikes may be undocumented. The absolute abundance of sperm whales in an area of 52933 km^2^, covering the territorial waters of the Canary Islands, was estimated from 2668 km of acoustic line-transect survey using Distance sampling analysis. Data on sperm whale diving and acoustic behaviour, obtained from bio-logging, were used to calculate g(0) = 0.92, this is less than one because of occasional extended periods when whales do not echolocate. This resulted in an absolute abundance estimate of 224 sperm whales (95% log-normal CI 120–418) within the survey area. The recruitment capability of this number of whales, some 2.5 whales per year, is likely to be exceeded by the current ship-strike mortality rate. Furthermore, we found areas of higher whale density within the archipelago, many coincident with those previously described, suggesting that these are important habitats for females and immature animals inhabiting the archipelago. Some of these areas are crossed by active shipping lanes increasing the risk of ship-strikes. Given the philopatry in female sperm whales, replacement of impacted whales might be limited. Therefore, the application of mitigation measures to reduce the ship-strike mortality rate seems essential for the conservation of sperm whales in the Canary Islands.

## Introduction

Worldwide, the anthropogenic impact on marine life is increasing due to an intensifying utilisation of the marine environment. During the last few decades maritime traffic volume and vessel speed have grown rapidly, leading to an increase in ambient noise [1], as well as in the number of ship-strikes [2]. The increase in the number of ship-strikes has been identified as of particular concern for species that spend long periods of time near the surface [3, 4] where they are vulnerable to ship-strikes. Cetaceans must come to the surface to breathe and consequently there are numerous documented cases of both serious injuries and mortalities caused by ship-strikes in cetacean populations [2, 5]. As a result, the mortality from ship-strikes has become an important conservation issue for cetaceans worldwide.

Although a variety of sailing and small motorised vessels have been reported as being involved in collisions [6], the occurrence and severity appears to increase with both the size and the speed of vessels [2, 5, 7]. Furthermore, the likelihood of collision increases in areas where high volumes of maritime traffic overlap with areas frequented by cetaceans [2]. Reports of ship-strikes include collisions with both small and large whales [8], but whales that swim slowly and spend long periods at shallow depths seem to be particularly vulnerable. To date, ten species of shallow-diving baleen whales have been reported to be globally involved in ship-strikes [2, 9]. Ship-strikes are of particular concern for small or isolated populations in near-shore habitats with high levels of maritime traffic [10, 11]. This is the case with the North Atlantic right whale (*Eubalaena glacialis*), where collisions with vessels have been identified as the main factor underlying the risk of extinction [12]. In general, the low reproduction rate of cetaceans, often compounded by complex social structures, can cause ship-strikes to have a significant impact at a population level [12,13].

To date, much of what is known about ship-strikes comes from coastal shallow-diving species [e.g. 2, 11]. In order to better understand the impact of ship-strikes on deep-diving species this study examines the sperm whale (*Physeter macrocephalus*) within an area that allows the assessment of the study's two essential objectives: the determination of the abundance of sperm whales in the area and a measure of the minimum ship-strike mortality rate. These objectives were feasible because of the proximity of the whale's deep-water habitat to land within the Canary Islands study area. The Canary Island archipelago consists of seven main volcanic islands, located off the northwestern coast of Africa.

Within the archipelago high densities of whales and dolphins coincide with areas of relatively high maritime traffic levels [14, 15, 16]. From 1991 to 2007 a total of 59 strandings, some 11% of the total number of strandings, were reported as showing signs of ship-strike. Eight different cetacean species could be identified: sperm whales (n = 24, 41%), pygmy sperm whales (*Kogia breviceps*, n = 7, 12%), Cuvier´s beaked whales (*Ziphius cavirostris*, n = 7, 12%), short-finned pilot whales (*Globicephala macrorhynchus*, n = 6, 10%), Gervais´ beaked whales (*Mesoplodon europaeus*, n = 1, 2%), fin whales (*Balaenoptera physalus*, n = 2, 3%), Bryde´s whales (*Balaenoptera brydei*, n = 2, 3%), and sei whales (*Balaenoptera borealis*, n = 1, 2%), in the remaining 9 strandings (15%) the species was not identified [15]. These reports of whales with signs of ship-strike increased in 1999 and have remained consistently high since [15]. This increase corresponds with the concurrent rise in the mean speed of ferries and the number of ferry journeys in the archipelago [14, 17]. Sperm whales are found year-round in the waters off the Canary Islands [18] and within the islands comprise the majority of stranding records where signs of ship-strike are present [15]. Since 1999 an average of two sperm whales per year have stranded within the archipelago with signs of ship-strike [15]. The sperm whale is listed as Vulnerable by the International Union for Conservation of Nature (IUCN) and is included in Annex IV of the European Union Habitats Directive.

In the Canary Islands collisions may be caused by international shipping, most often transiting through the two International Maritime Organization (IMO) designated shipping lanes within the Particularly Sensitive Sea Area (PSSA) of the Canary Islands [19], and by inter-island vessel traffic. Ferries operating in the Canary Islands usually travel at speeds of 23 to 40 knots (some 43 to 74 km/h) [16]. Laist *et al*. [2] and Vanderlaan and Taggart [5] correlated increasing vessel speed (above 10 knots) with a greater probability of ship-strikes and of whales sustaining a lethal injury from such strikes.

Here we provide an estimate of the absolute abundance and density of sperm whales within the Canary Islands and a snap-shot of their distribution. The results are used to assess the potential for ship-strikes in the archipelago to impact on the local abundance of sperm whales. This assessment shows that in the long term this deep-diving species cannot sustain the current level of ship-strikes within this local area. This has implications for other areas, particularly remote offshore areas, where high concentrations of deep-diving whales may coincide with high levels of ship traffic resulting in the impacts of ship-strikes going unnoticed.

## Materials and Methods

### Survey area and transect design

**Fig 1:**
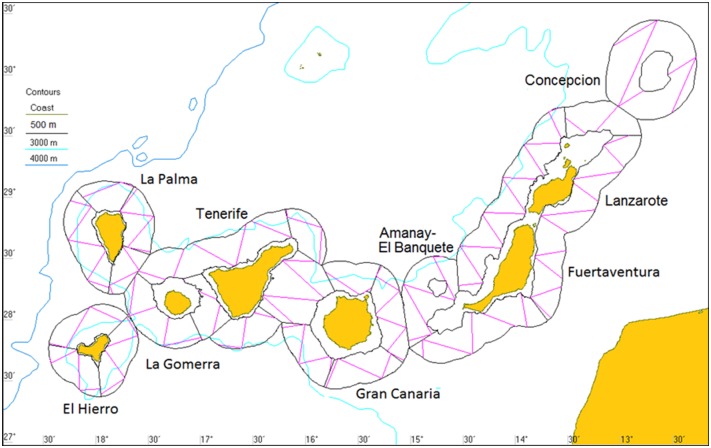
Survey area. Survey area indicating the names of the islands and seamounts, and showing selected isobaths, the survey blocks (black lines) and the designed transects (magenta lines).

The survey was designed to cover an area extending from the islands' shelf-edge, across the slope and into abyssal waters; the 500 m isobath was used as the inner survey boundary while the outer boundary was 27 km offshore from this (Fig 1). This survey area included the territorial waters around the Canary Islands, where the Spanish Government has a mandate from national and international conservation law to manage protected species and mitigate anthropogenic impacts for conservation purposes. Furthermore, the survey area encompassed the Concepcion, Amanay and El Banquete seamounts (Fig 1), which are proposed for inclusion in the EU Nature 2000 network of protected areas. The total area of the zone surveyed was 52933 km^2^. The zone was divided into 23 survey blocks to facilitate transect design (Fig 1), which was carried out using *Distance* version 6.0 release 2 (distancesampling.org, [20]. An equidistant zigzag transect design was used, with a random start to the zigzags within each block, and with a similar coverage rate across all blocks; this resulted in 99 transects totalling 3030 km in length. The principal axis for the zigzag design within each block was as close to parallel to the bathymetry as possible, resulting in transects approximately perpendicular to the slope. The average survey speed was 6.4 knots (11.8 km/h), essentially fulfilling the recommendation ([21] p251) that surveys should be conducted at speeds of at least 2–3 times that of the animals' speed in order to avoid an overestimation of animal density; typical speeds for sperm whales are 1.9–3.2 knots (3.5–6 km/h) [22]. The survey was carried out with the permission of the Canary Islands Government, and since the survey methodology was passive, did not require permitting from ethical committees.

Due to weather conditions and logistical reasons the survey was performed over three separate time periods: (i) 24^th^ September to 18^th^ October 2009: the transects around the Concepcion seamount, Lanzarote, Fuerteventura, La Palma and El Hierro, as well as part of Gran Canaria, Tenerife, La Gomera, Amanay and El Banquete seamounts were surveyed; (ii) 6^th^-16^th^ December 2009: the transects around Tenerife and La Gomera were completed; (iii) 23^rd^-26^th^ February 2010: the transects around Gran Canaria, Amanay and El Banquete seamounts were completed (Fig 1).

### Acoustic Survey

Acoustic data were collected using a hydrophone array with two broadband frequency hydrophone elements (HP-03) 23.4 cm apart and a broadband preamplifier with a 2 kHz high-pass filter (Ecologic, J. Gordon) towed 200 m behind the vessel. The hydrophone was connected to an amplifier box built by Seiche Measurements Ltd. Sound was digitised with an analogue to digital converter (ADC *E-MU Tracker Pre*) at a sampling rate of 96 kHz and recorded continuously as WAV files using IFAW's *Logger* software, version 4.05.0002. An automatic click detection and classification program, *Rainbow Click* [23] version 4.07.0002, ran continuously throughout the survey. *Rainbow Click* identified candidate sperm whale clicks and calculated the bearings to these clicks in real-time by measuring the difference in time of arrival of the signal at the two elements.

### Click Analysis

To obtain perpendicular distances to whales it was necessary to use the bearings to clicks produced by individual whales and to intersect these as the vessel passed the whales. Clicks were assigned to individual whales during post-processing by analysing the WAV recordings using *Rainbow Click* [24]. The program identified candidate sperm whale clicks and plotted these on bearing against time, amplitude against time and inter-click interval against time plots. The program also provided plots of the waveform and the power spectrum for individual clicks and allowed playback of selected clicks. An analyst used these tools to assign clicks to click trains and click trains to individual whales. Clicks of individual sperm whales appeared as sequences of regularly spaced clicks on a bearing that changed slowly with time as the vessel passed the animals.

### Determining Perpendicular Distances

A routine for estimating whale positions, implemented in Matlab 6.5 (www.mathworks.com), was used to estimate the position of each whale and consequently its perpendicular distance to the track-line. The routine combined data for the ship's course with data on bearings to clicks from individual whales and, by simplifying the situation and assuming whales were at the sea surface, crossed these bearings at the surface. This produced two areas of intersection, one to the left and one to the right of the track. The routine selected the side with the most coherent intersection of bearings and the distance between this point and the survey was then used in the program *Distance*. The simplification of assuming whales were at the surface means that for diving whales the perpendicular distance is over-estimated. The bias introduced by this simplification was investigated by Leaper *et al*. [25] and found not to be significant.

### Line-transect g(0) estimation

Conventional Distance sampling (CDS) analysis [21] was used to calculate the density of whales in the survey area. A detection model was used to calculate the equivalent width of the survey strip as if a strip-transect survey (i.e. of fixed width) had been used, this is termed the effective strip half-width (ESHW). This allows the density of animals within the effective strip to be estimated. Hence, density estimates rely on the knowledge of a detection function, i.e. the probability of detecting an animal as a function of its perpendicular distance from the track-line. Here, a key parameter is g(0), the probability of detecting animals at zero horizontal distance from the track-line, which is assumed to be certain. However, sperm whales occasionally spend extended periods at the surface, resting and/or socialising, when they do not echolocate, this can result in g(0) being less than one. Previous studies have used g(0) = 1 as an approximation to the actual value of g(0) as evidence suggested that extended non-echolocating periods were relatively rare (e.g. [26]), and furthermore accurate data on the extent and frequency of such periods was either not available or not easily obtained.

The probability of acoustically detecting whales is a combination of the probability of whales vocalising and the probability of detecting these sounds. Following Barlow *et al*. [27] we assumed that, within the ESHW either side of the track-line, the availability bias, resulting from animals being missed because they were not vocalising, dictates g(0). We therefore estimated the acoustic availability bias of sperm whales using a Monte Carlo simulation. The model used defines a ship travelling through a 1-D model space (the transect line) of *L* km length (randomly chosen between 100 and 1000 km) with a fixed speed. *N* (1–300) randomly distributed stationary whales were placed on the track-line changing their detectability (time echolocating/not echolocating) after a period t_e_/t_ne_. Periods t_e_ and t_ne_ were randomly chosen from a distribution of t_e_ and t_ne_ values derived from empirical data (see below). Whales vocalising within a range equal to the ESHW behind or ahead of the travelling vessel were considered as detected. We used the ESHW obtained from the perpendicular distances as a proxy for the ESHW ahead and behind the vessel, this can cause a bias [28], however due to the long-range detection of sperm whales' echolocation clicks and the relatively short duration of the non-echolocating periods this bias can be considered negligible.

**Fig 2:**
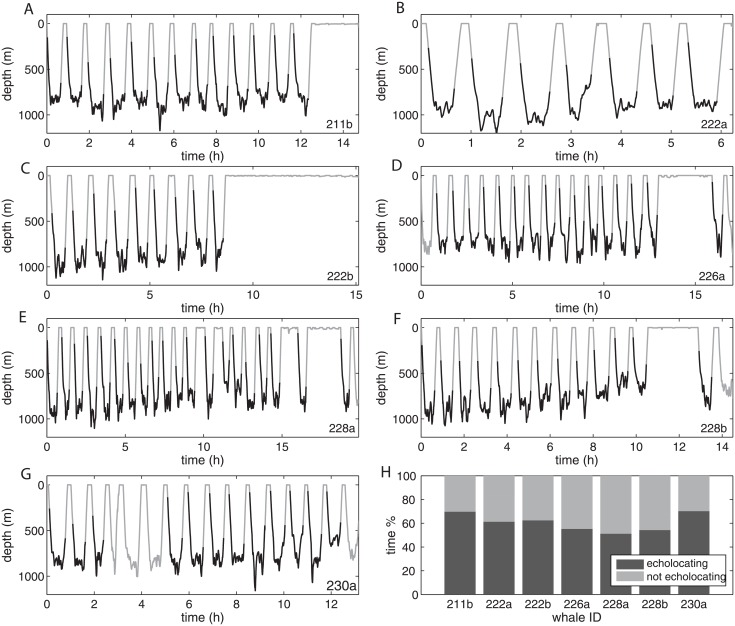
Dive profiles and time budgets. Dive profiles of each sperm whale tagged off the Azores in 2010, A) 211b, B) 222a, C) 222b, D) 226a, E) 228a, F) 228b and G) 230a. Black lines mark the echolocation periods of the dives. Dives without echolocation periods were not used for the analysis because they were incomplete due to tag release (226a, 228a, 228b and 230a) or the echolocation period could not be identified due to errors in the original sound files (226a and 230a). H) Pooled time budget of the seven sperm whales tagged off the Azores. The bars show the periods of time spent: (i) not echolocating (grey) and (ii) echolocating during deep dives (black). Whale codes are as for Table 1.

For animals on the track-line the survey’s detection time-window is defined as 2 × detection range / survey speed. We estimated g(0) by dividing the number of detected whales *n* by the total number of whales present in the model space: *g(0) = n/N*. The distributions of t_e_ and t_ne_ values were derived from the number and duration of the periods that sperm whales spend echolocating or not, respectively. This was estimated using data from acoustic and movement tags (DTag, [29]) deployed on seven sperm whales off the Azores; this provided 94 hours of combined acoustic and movement data (Fig 2). Three of the seven sperm whales were recognised as females, given that calves were seen suckling from them; the rest may have been adult females or immature males, both similar in appearance (Table 1).

**Table 1:** Information on the sperm whale DTag recordings. Whale codes are generated by combining the three-digit Julian day and the deployment order of the tag in that day (as a letter). A complete dive cycle comprises a foraging dive and the following inter-dive interval. Only complete dive cycles were used here, and the first dive cycle after tagging was excluded. Foraging dives were divided into an echolocation period (when animals were producing 'usual' clicks [30]) and periods of silent descent and ascent (between the surface and the depth at which animals started and stopped clicking). Durations are given as the median of each period (range).

Whale code	Gender	# dive cycles	Echolocation period (minutes)	Periods of silent descent and ascent (minutes)	Inter-dive interval (minutes)
211b	female or immature male	11	41.1 (32.8–45.1)	8.8 (7.9–11.1)	8.5 (7.2–9.8)
222a	female	5	32.7 (25.4–36.9)	11.8 (10.1–10.2)	8.3 (7.5–11.5)
222b	female or immature male	7	37.0 (32.7–39.9)	9.1 (8.8–12.1)	12.0 (9.5–16.8)
226a	female	16	33.1 (28.9–38.4)	6.3 (5.1–9.9)	9.4 (8.0–175.0)
228a	female or immature male	19	30.7 (24.8–35.5)	7.2 (5.6–10.7)	9.9 (8.7–126.6)
228b	female or immature male	12	35.7 (29.1–40.8)	9.5 (5.9–9.8)	9.9 (8.9–139.4)
230a	female	11	35.2 (27.1–38.6)	6.3 (5.0–10.8)	7.9 (6.4–9.6)

**Fig 3:**
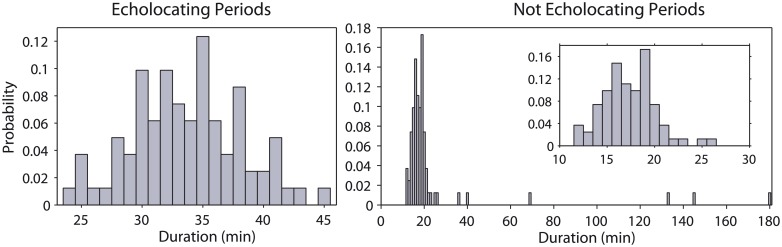
Probability histograms. Probability histograms of the duration of echolocating and not echolocating phases (in 1 minute-bins) for the seven sperm whales tagged off the Azores. The echolocation phase represents the time elapsed from the start to the end of ‘usual’ clicking in a foraging dive; the silent phase, i.e. when the whale was not echolocating, is the sum of the silent descent, silent ascent and the time spent at the surface between dives (IDI). The simulation model was run with parameters chosen from these echolocating/not echolocating distributions.

The dive profiles of tagged whales were divided into dive cycles comprising a foraging dive and the following inter-dive interval (IDI). Only complete dive cycles (n = 80) were used and the first dive cycle after tagging was excluded from the analyses to reduce potential bias due to the reaction of whales to tagging. We considered that whales were available for acoustic detection only during the echolocation periods within foraging dives, because only ‘usual’ echolocation clicks, and not social sounds such as codas, were used in our survey to detect and localise whales. Hence, the vocal period over which sperm whales were acoustically detectable was defined as the interval between the beginning and the end of clicking within a dive. We pooled data from all whales to estimate the probability of a sperm whale being available for acoustic detection (Fig 3).

An estimate of g(0) was calculated for our ESHW of 4.168 km and survey speed of 6.4 knots. Additionally the simulation model was run for a representative range of other ESHWs and survey speeds. For each combination of parameters the simulation was run 2000 times (S1 File).

### Abundance estimation

The ESHW was estimated from acoustically derived perpendicular distances of the whales from the track-line using the software Distance, version 6.0 release 2 [20]. Given that sperm whale groups can extend across relatively large areas [22], distances to individual animals were used as the sampling unit, rather than using distances to the centres of groups and the number of whales within each group [see Lewis *et al*. in prep]. Sperm whale density ($\hat{D}$) was estimated using the following equation:

$$\hat{D}=\;\frac{n}{2\hat{\mu }L}\times \frac{1}{g\left(0\right)}\;\left[\text{whales}/\text{unit area}\right]$$ (1)

where *n* is the number of sperm whales detected acoustically, $\hat{\mu }$ is the ESHW, *L* is the total surveyed transect length and g(0) is the estimated fraction of whales that were detected along the transect. Rather than assume g(0) = 1 we used our estimation of the value of g(0). The estimate of abundance $\hat{N}$ in the surveyed area *A* is given by:

$$\hat{N}=A\times \hat{D}$$ (2)

### Overlapping effort at transect corners

Where transects meet, or nearly meet, at the corners of the zigzag design, there may be an overlap in the area surveyed. Where such transects are surveyed consecutively this may result in animals in this area being counted twice. Such detections will generate some non-independence when transects are used as the sampling unit for variance estimation. To address this situation whales were only counted once in these areas. To compensate for the consequent reduction in effort the sum of such overlapping areas was calculated and a correction applied to the density estimate within the program *Distance*.

### AIS data

The transmission of AIS (Automatic Identification System) data is mandatory for large vessels. A sample of AIS data collected by KAI Marine Services in the framework of the LIFE+INDEMARES Project was made available for this study to obtain a representative distribution of ship traffic in the waters off the Canary Islands. It was not the objective of this to obtain an absolute measure of the number of ships crossing the archipelago. For this analysis AIS data from a 28 day period were used. These comprised of data from seven days in each of the months of January, April, July and October chosen randomly from a data set of 365 days of AIS signals. The AIS data layer was overlaid with a grid of cell size 5'x5' and the number of AIS signals present by unit area (km^2^) was estimated assuming that the AIS data analysed here were representative of the shipping traffic thorough the year. Thus for each grid cell we estimated a mean value of AIS/km^2^/year. No information about vessel type or cargo was obtained from the AIS data.

## Results

### Line-transect g(0) estimation

The diving behaviour of the tagged whales was consistent with the highly stereotyped behaviour described by Watwood *et al*. [31] for sperm whales in low latitudes (Fig 2). Whales started clicking on average at 200 m (61–452 m) depth and produced echolocation clicks almost continuously until they stopped searching for prey at a mean depth of 615 m (359–806 m) at the beginning of the ascent from their dives. Whales spent on average 60% of their time echolocating, with echolocation periods lasting on average 34 min (25–45 min, Fig 3). Periods not echolocating, i.e. the sum of the silent descent stage, the silent ascent from a dive and the inter-dive interval, lasted a mean of 24 min (12–181 min, Fig 3). The Monte Carlo simulation estimated the probability of detecting sperm whales on the transect line g(0) = 0.92 (σ = 0.031) for this survey (S1 Fig). The results for other survey settings are presented in the supplementary material (S1 File).

### Abundance estimation

**Fig 4:**
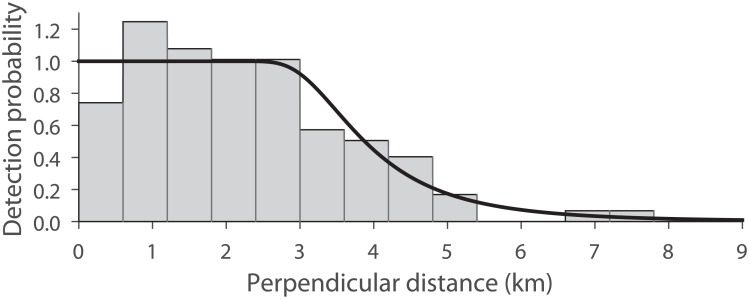
Histogram and detection function. Histogram of perpendicular distances to sperm whale acoustic detections and the fitted detection function. Data were grouped into 600 m bins for the histogram, although exact distances were used to fit the detection function. Data were truncated at 9 km.

During 225 hours of acoustic effort covering 2668 km of track-lines, perpendicular distances from the track were determined to 85 acoustically detected sperm whales; the maximum perpendicular distance was 11 km. Acoustic detections were truncated at 9 km, resulting in distances to 83 sperm whales being used to fit a detection function. Based on the minimum Akaike Information Criterion (AIC), the Hazard Rate Key model with no adjustment terms and no constraints was selected (Fig 4), resulting in an ESHW of 4168 m (95% log-normal CI 3599–4826). The total area of overlapping effort at consecutively surveyed zigzag corners was calculated as 4.2% of the total area surveyed and this was included in the density calculation.

The abundance estimate for the number of sperm whales in the survey area before applying our estimate of g(0) is provided here to allow for the substitution of alternative values of g(0). This estimate (assuming g(0) = 1) was 206 animals (CV of 32.0% and 95% log-normal CI 111–383) with an estimated density of 3.90 whales/1000 km^2^ (95% log-normal CI 2.10–7.242.09–7.22). Applying our estimate of g(0) and σ within *Distance* resulted in an absolute abundance estimate of $\hat{N}$ = 224 sperm whales (95% log-normal CI 120–418) within the survey area, with a corresponding density estimate of $\hat{D}$ = 4.24 whales/1000 km^2^ (95% log-normal CI 2.27–7.89). The CV for the abundance and density estimates was 32.2%.

### Sperm whale distribution

**Fig 5:**
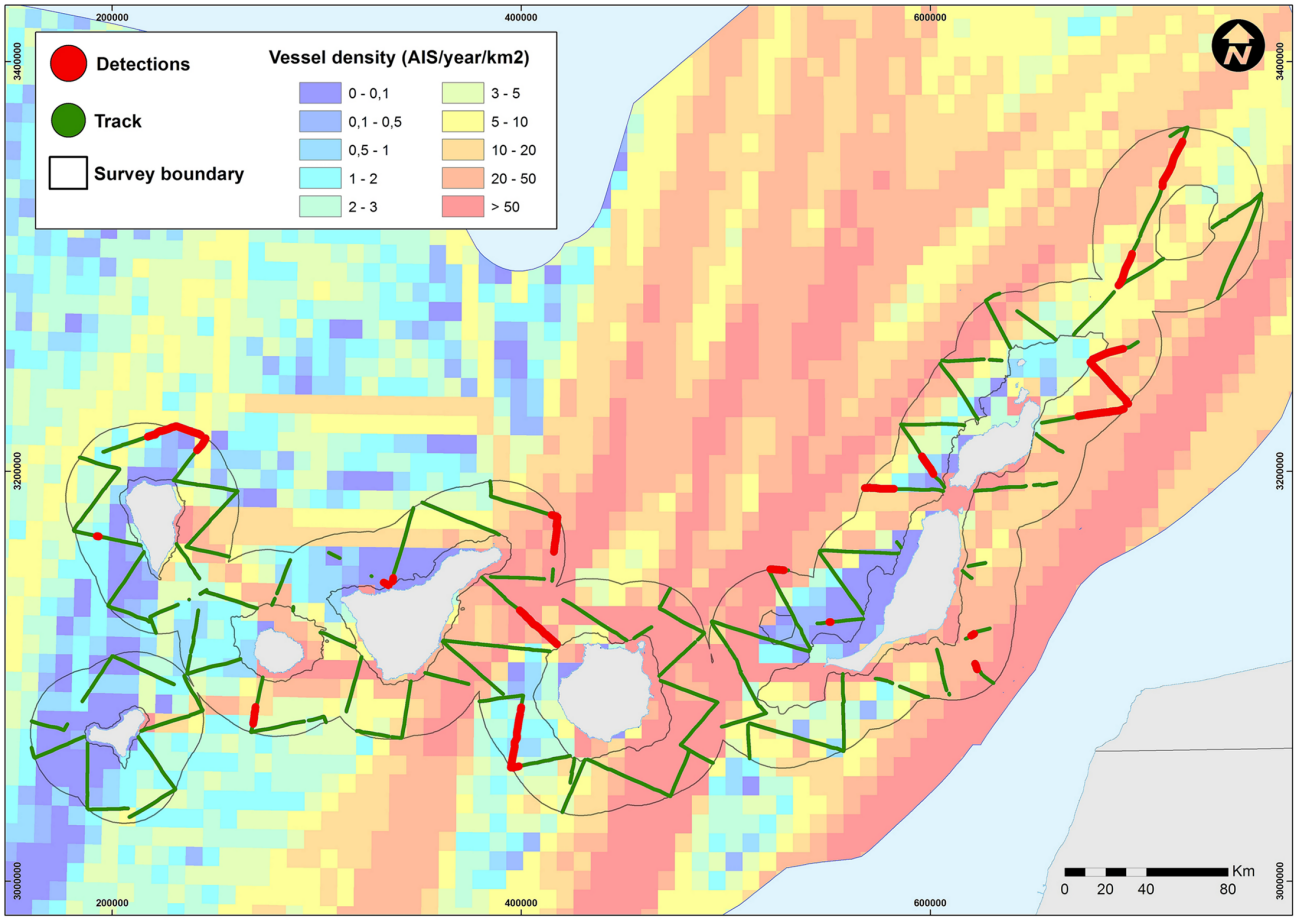
Sperm whale survey effort and detections overlaid on map of marine traffic density. Acoustically surveyed tracks are shown as green lines, while tracks in red show when sperm whales were acoustically detected. Marine traffic data were obtained from AIS data and coloured by traffic density (given as AIS signals/km^2^/year).

Sperm whales were distributed throughout the archipelago (Fig 5); there were detections around all surveyed seamounts and all major islands, except for El Hierro. Most detections were made in the following areas: the channel between Tenerife and Gran Canaria, north of La Palma, east and west of the channel between Fuerteventura and Lanzarote, and in the vicinity of the Concepcion seamount (Fig 5). There was a significant concentration of whales in a relatively small area to the north of La Palma, where four groups were detected, one of which contained over 20 individuals. Following this, the area with the highest number of groups was the channel between Tenerife and Gran Canaria. In general there was an increase in the number of detections with increasing depth within the surveyed area.

## Discussion

Knowledge of distribution and abundance is essential for effective management towards conservation of wildlife. Here, we estimate the absolute abundance and density of sperm whales in the Canary Islands and provide a snap-shot of their distribution. The results are used to assess the sustainability of ship-strikes for sperm whales in the archipelago and suggest that the Canary Islands might be acting as an attractive sink habitat.

### Detection function

The estimate of g(0) for our survey was 0.92. This means that sperm whale abundance would be underestimated by approximately 8% should we fail to account for animals not echolocating for periods exceeding our survey's detection time-window of 43 minutes. This time-window is given by 2 × detection range/survey speed. Here we use the ESHW of 4.2 km as an approximation to the detection range, and use the mean survey speed of 11.8 km/h. The estimate of g(0) was performed using bio-logging data from the Azores, on the basis that sperm whales will behave (Fig 2) similarly in the Azores and the Canary Islands given both regions have similar habitats and lie within the Macaronesia ecoregion [32]. Moreover, the diving and vocal behaviour of sperm whales across warm and temperate regions has been shown to be highly stereotyped [31].

The ESHW of 4.2 km for this survey was about half that of the ESHWs estimated for surveys performed in comparable latitudes, where groups comprising of females and immature animals predominate, e.g. 8–10 km [33, 34]. This difference is probably due to masking caused by elevated levels of noise generated by our survey vessel. The histogram of perpendicular distances (Fig 4) shows a dip close to the track line (<600 m) with a peak further out (600–1800 m). This dip and peak in the histogram is likely to have resulted from the process of rotating the distances to dived whales to the surface in order to obtain horizontal perpendicular distances for Distance analysis (as described in Materials and Methods). Leaper *et al*. [25] showed that the bias introduced by this simplification was small (e.g. <1% for animals diving to 1000 m), furthermore the Hazard Rate Key model, aided by a plateau in the histogram data beyond the peak, effectively smoothed out this perturbation.

### Abundance estimation & distribution

The density of sperm whales within the survey area was 4.24 whales/1000 km^2^ (CI 2.27–7.89), just 18% of the density estimate obtained during the first survey of sperm whales in the Canary Islands, made in 1995, of between 19.6 and 27.7 whales/1000 km^2^ [18]. This reduction is considerable, but should be interpreted with caution, given that both studies differ in several respects: (i) our survey was performed in autumn-winter, while André surveyed in summer; (ii) André covered a smaller survey area—extending to 20 km offshore (ours extended to 27 km from the 500 m isobath); (iii) André used acoustic point sampling, whereas we used line transect sampling, and iv) André used a mean value for group size in the abundance estimation, based on observations of sperm whales off the central islands, whereas we used distances to each animal detected within a group for CDS analysis.

Although sperm whales exhibit a nomadic ranging behaviour [35], and consequently can be found throughout the Canary Islands, we also found that there were specific areas where sperm whales were concentrated, and that many of these areas were consistent with those reported by [18]. These areas included: the area north of La Palma, the channel between Gran Canaria and Tenerife, the area southwest of La Gomera, the areas southeast and northwest of Fuerteventura, and the area northeast of Lanzarote (Fig 5). Additionally, a new area of aggregation was found around the Concepcion seamount, an area not surveyed by André. While these two surveys only constitute snapshots of the distribution of the whales, the coincidence of areas with higher whale concentrations in both surveys, which were carried out more than a decade apart, suggests that sperm whales may have preferred habitats in the archipelago.

### Potential ship-strike risk

This study suggests that sperm whales may occupy preferred areas within the archipelago, which might be quite stable across time, likely indicating important foraging habitats and some degree of site fidelity within the region. Some of these aggregation areas coincide with areas of high maritime traffic density [16, 36], creating areas with an elevated ship-strike risk (Fig 5). For example, the preference of sperm whales for the channel between Tenerife and Gran Canaria may explain the high number of strandings with signs of ship-strikes recorded in Tenerife [15, 16]. This channel is crossed by inter-island ferry routes which are used by fast (travelling at 23–25 knots) and high speed (travelling at 30–40 knots) ferries some 130 times per week [16]. A shipping lane endorsed by the IMO also passes through the channel (Fig 5). The dominant trade-wind and marine currents in the archipelago are from the northeast and therefore favour the stranding of carcasses on the island on the western side of the channel, i.e. Tenerife.

Carrillo and Ritter [15] showed that in the Canary Islands at least two sperm whales per year died between 1999 and 2007 with signs of ship-strike. In general, caution is required when inferring ship-strike as the cause of a stranding, given that injuries may be post-mortem, potentially resulting in an overestimation of strike-related mortalities [2]. However, all necropsies (n = 17) performed on sperm whales that stranded with signs of collision in the Canary Islands confirmed ship-strike as the cause of death [37, 38]. Necropsies were performed on animals stranded in an adequate state of conservation, independently of the cause of death. This strongly suggests that the estimate of annual ship-strike mortality rate gathered from strandings is not overestimated. In fact, the actual number of ship-strike mortalities is likely to be underestimated by stranding data, since not all carcasses strand and not all stranded animals are discovered. For example, Williams *et al*. [39] suggested the rate of discovery of sperm whale carcasses in the Gulf of Mexico was only 3%. This rate may be higher in the Canary Islands due to the proximity of deep waters to the shore; however, the steepness of the coast may impede the discovery and recovery of stranded animals in many areas [40]. Furthermore, when carcasses of stranded whales are found, signs of collision may not be detected or conclusive.

The average number of strandings per year showing signs of collision represents about 1% of the 224 sperm whales estimated to be within the surveyed waters around the archipelago. Whitehead [41] estimated the maximum rate of increase of a sperm whale stock (*r*) to be about 1.1% per year. Applying this to our abundance estimate gives a maximum rate of increase of 2.5 whales per year, a value that is very close to the 2 whales that strand per year with signs of collision. Moreover, given the likelihood that strandings are underestimating the actual ship-strike mortality rate, mortalities due to ship-strikes would likely exceed the reproduction rate of the number of sperm whales surveyed in the Canary Islands. Nevertheless, the overall effect of anthropogenic mortality on an animal population will depend on whether or not the losses can be compensated for by external recruitment [42]. The lack of data on the site fidelity and the connectivity of sperm whale groups inhabiting the Canary Islands preclude an accurate evaluation of the risk posed by ship-strikes for this pelagic deep-diving species in the archipelago. However, given female philopatry [43] and the complex social structure of this cetacean species [44, 45], the fact that ship-strikes in the Canary Islands appear to preferentially affect females and young animals [15] may increase the risk of population level effects.

This study presents the first attempt to assess the potential effects of ship-strikes on sperm whales in the Canary Islands, and represents the best available information on which to base management actions until further data on population structure becomes available. The results suggest that the archipelago could be considered as an attractive sink habitat [42]. Such a habitat is defined as a high-quality area in terms of occupancy (e.g. for food resources), but low-quality in terms of poor survival rates arising from human activities [46]. When such apparently attractive sink habitats are utilised in preference to other habitats, the population as a whole may decline or become locally extinct [47]. The implications of selecting an attractive sink habitat for a population´s long term survival will depend on the population size [48], the strength and flexibility of the habitat selection [49]) and the amount of sink habitat in the landscape [42]. In the face of uncertainty, it is important to implement precautionary approaches to management [50, 51]. Applying the precautionary approach in this case would mean reducing the anthropogenic impact, i.e. that caused by ship traffic, to reduce human induced mortality in an apparently high-quality habitat for sperm whales in the North Atlantic.

Sperm whales in the Canary Islands are not well studied, but it is recognised that sperm whales occur mainly in groups composed primarily of females and their offspring which are present in the islands all year-round [18]. Sperm whales in the North Atlantic are considered a single stock [52], and genetic studies carried out by Alexander *et al*. [53] show an mtDNA differentiation between the Canary Islands and the western North Atlantic, which suggests that there is no apparent connection between females groups within the west and east North Atlantic Ocean. Given the local philopatry of females ([43, 53], previous studies have suggested that management of sperm whales should be focused on females and immature components of the population [53, 54, 55]. Here, we support the idea that mitigation measures should be implemented in the archipelago in order to reduce ship-strike mortality rates. Mitigation measures, proposed by Silber *et al*. [56] and Carrillo and Ritter [15], should be evaluated for their effectiveness and be implemented for both the inter-insular ferries and the international shipping in collaboration with the IMO. In this study, we focused on the sperm whale, however all cetacean species involved in ship-strikes in the Canary Islands [15, 37] will potentially benefit from these mitigation measures.

## Supporting Information

S1 Fig Estimates of g(0). Estimates of g(0) for acoustic line transect surveys of female/immature sperm whales as a function of vessel speed and effective strip width. The black cross marks the ESHW (4.168 km) and survey speed (6.4 knots) for this survey, resulting in an estimated g(0) of 0.92.
(EPS)

S1 File Line-transect g(0) estimaions. (DOCX)

S2 File Data imported into Distance program. (DOCX)

S3 File Data imported into simulation model. (DOCX)
