# Correction: Abundance and Distribution of Sperm Whales in the Canary Islands: Can Sperm Whales in the Archipelago Sustain the Current Level of Ship-Strike Mortalities?

**DOI:** 10.1371/journal.pone.0155199

**Published:** 2016-05-04

**Authors:** Andrea Fais, Tim P. Lewis, Daniel P. Zitterbart, Omar Álvarez, Ana Tejedor, Natacha Aguilar Soto

There are errors in the Funding section. The correct funding information is as follows: Funding was provided through an agreement between the Canary Islands Government and the Spanish Ministries of the Environment and Defence. Additional survey effort on the Amanay, Banquete and Concepción seamounts was funded by the Fundación Biodiversidad-MAGRAMA via the LIFE-INDEMARES project. OA is employed by a commercial company, CIMA Canarias, and received support in the form of a salary. The specific role of this author is articulated in the Author Contributions section. NAS was funded during data collection of this work by the EU-FP7 Marie Curie project SOUNDMAR, and she is currently funded by project ECOSOUND within the Horizon 2020 EU program. Additional data analysis of this work was funded by Fundación Biodiversidad-MAGRAMA via the project Canarias con la Mar. These funders did not have any additional role in study design, data collection and analysis, decision to publish, or preparation of the manuscript.
