# Supplementary material for: Abundance and Distribution of Sperm Whales in the Canary Islands: Can Sperm Whales in the Archipelago Sustain the Current Level of Ship-Strike Mortalities?
Source: PLoS One. 2016 Mar 21;11(3):e0150660. doi: 10.1371/journal.pone.0150660 (PMC4801403; doi:10.1371/journal.pone.0150660)
Supplement: S1 File — (DOCX) [file pone.0150660.s002.docx]

**S1 File. Line-transect g(0) estimations**

Estimated values of g(0) for our model depend on the length of the acoustic detection time-window, with longer time-windows producing higher values of g(0) and shorter time-windows producing lower values. The length of the time-window in turn depends on both the survey speed and the detection distance (as measured by the effective strip half-width (ESHW)) with the length of the window increasing with slower survey speeds and larger ESHWs, and decreasing with higher speeds and smaller ESHWs; as the time-window shortens so the probability that a whale on the track-line will pass across the detection time-window without echolocating (and be missed) will increase. It should be noted that slower survey speeds, while increasing the detection time-window, can introduce a bias arising from animal movement, and it is therefore recommended that survey speeds should be at least two or three times faster than that of the average speed of the animal in order to avoid this bias [21]. Whitehead [57] reported an average speed for female and immature sperm whales of 2.05 knots suggesting survey speeds should therefore be no slower than 4.1 knots and ideally faster than 6.2 knots. As the detection time-window increases then the chance of not detecting a whale on the track-line due to it being silent during the relatively short quiet phase of a normal dive cycle quickly diminishes to zero, however the smaller component of chance of not detecting a whale arising from it being silent during the much less frequent but longer periods of silence associated with resting/socialising periods diminishes more slowly, with a small contribution persisting even at larger detection time-windows (e.g. at an ESHW of 15 km and slow survey speeds). While sperm whales may not produce echolocation clicks during the resting/socialising periods they may produce codas [58] or chirrups [30]. However these communication clicks have relatively short detection ranges and are often produced irregularly making them difficult to use to reliably locate individual animals, consequently only echolocation clicks were used to determine detection distances to animals in this survey.

**Table A. Simulation outputs.** Simulation outputs for different survey speeds and effective strip half-widths used in estimating track-line detection probabilities of female/immature sperm whales.

| Strip width (km) | Survey speed (knots) | | | | | | | | | | | | | | | | | | | |
| --- | --- | --- | --- | --- | --- | --- | --- | --- | --- | --- | --- | --- | --- | --- | --- | --- | --- | --- | --- | --- |
|  | 1 | | 2 | | 3 | | 4 | | 5 | | 6 | | 7 | | 8 | | 9 | | 10 | |
|  | g(0) | g(0) sd | g(0) | g(0) sd | g(0) | g(0) sd | g(0) | g(0) sd | g(0) | g(0) sd | g(0) | g(0) sd | g(0) | g(0) sd | g(0) | g(0) sd | g(0) | g(0) sd | g(0) | g(0) sd |
| 0.5 | 0.911 | 0.036 | 0.838 | 0.047 | 0.751 | 0.065 | 0.707 | 0.060 | 0.680 | 0.065 | 0.662 | 0.059 | 0.650 | 0.068 | 0.641 | 0.069 | 0.634 | 0.063 | 0.628 | 0.066 |
| 1.0 | 0.939 | 0.033 | 0.910 | 0.040 | 0.889 | 0.042 | 0.835 | 0.051 | 0.787 | 0.057 | 0.751 | 0.056 | 0.725 | 0.057 | 0.707 | 0.055 | 0.697 | 0.062 | 0.681 | 0.061 |
| 1.5 | 0.961 | 0.024 | 0.927 | 0.035 | 0.908 | 0.038 | 0.896 | 0.048 | 0.875 | 0.048 | 0.836 | 0.051 | 0.800 | 0.052 | 0.771 | 0.057 | 0.748 | 0.058 | 0.732 | 0.062 |
| 2.0 | 0.980 | 0.018 | 0.938 | 0.033 | 0.922 | 0.035 | 0.908 | 0.039 | 0.898 | 0.040 | 0.889 | 0.041 | 0.866 | 0.044 | 0.835 | 0.051 | 0.805 | 0.054 | 0.786 | 0.059 |
| 2.5 | 0.993 | 0.010 | 0.950 | 0.030 | 0.929 | 0.040 | 0.917 | 0.038 | 0.907 | 0.039 | 0.901 | 0.036 | 0.892 | 0.040 | 0.880 | 0.041 | 0.858 | 0.048 | 0.835 | 0.046 |
| 3.0 | 0.998 | 0.005 | 0.960 | 0.025 | 0.938 | 0.030 | 0.923 | 0.037 | 0.916 | 0.032 | 0.907 | 0.039 | 0.900 | 0.037 | 0.895 | 0.039 | 0.890 | 0.042 | 0.871 | 0.044 |
| 3.5 | 0.997 | 0.010 | 0.969 | 0.022 | 0.946 | 0.028 | 0.931 | 0.031 | 0.921 | 0.036 | 0.912 | 0.036 | 0.905 | 0.041 | 0.899 | 0.039 | 0.894 | 0.038 | 0.887 | 0.041 |
| 4.0 | 0.997 | 0.010 | 0.979 | 0.018 | 0.952 | 0.027 | 0.936 | 0.033 | 0.927 | 0.032 | 0.919 | 0.034 | 0.909 | 0.040 | 0.905 | 0.041 | 0.900 | 0.040 | 0.894 | 0.048 |
| 4.5 | 0.997 | 0.012 | 0.986 | 0.015 | 0.959 | 0.027 | 0.941 | 0.036 | 0.930 | 0.033 | 0.922 | 0.035 | 0.916 | 0.036 | 0.907 | 0.048 | 0.902 | 0.041 | 0.900 | 0.043 |
| 5.0 | 0.997 | 0.006 | 0.990 | 0.012 | 0.963 | 0.028 | 0.948 | 0.029 | 0.936 | 0.031 | 0.924 | 0.035 | 0.919 | 0.036 | 0.913 | 0.045 | 0.910 | 0.037 | 0.902 | 0.039 |
| 5.5 | 0.997 | 0.010 | 0.993 | 0.010 | 0.970 | 0.020 | 0.951 | 0.030 | 0.941 | 0.027 | 0.931 | 0.031 | 0.922 | 0.035 | 0.917 | 0.037 | 0.911 | 0.036 | 0.913 | 0.038 |
| 6.0 | 0.997 | 0.006 | 0.995 | 0.009 | 0.976 | 0.018 | 0.955 | 0.030 | 0.944 | 0.029 | 0.927 | 0.034 | 0.927 | 0.033 | 0.921 | 0.034 | 0.915 | 0.038 | 0.911 | 0.036 |
| 6.5 | 0.997 | 0.009 | 0.994 | 0.012 | 0.981 | 0.018 | 0.960 | 0.025 | 0.948 | 0.032 | 0.938 | 0.028 | 0.930 | 0.030 | 0.924 | 0.037 | 0.918 | 0.034 | 0.913 | 0.035 |
| 7.0 | 0.997 | 0.007 | 0.994 | 0.012 | 0.985 | 0.015 | 0.966 | 0.021 | 0.951 | 0.029 | 0.941 | 0.032 | 0.932 | 0.036 | 0.926 | 0.034 | 0.922 | 0.035 | 0.915 | 0.035 |
| 8.0 | 0.997 | 0.007 | 0.995 | 0.009 | 0.989 | 0.016 | 0.974 | 0.022 | 0.959 | 0.025 | 0.947 | 0.029 | 0.941 | 0.030 | 0.932 | 0.033 | 0.926 | 0,034 | 0.920 | 0.037 |
| 9.0 | 0.997 | 0.008 | 0.995 | 0.010 | 0.992 | 0.013 | 0.982 | 0.018 | 0.966 | 0.021 | 0.953 | 0.028 | 0.944 | 0.029 | 0.937 | 0.032 | 0.930 | 0.038 | 0.926 | 0.033 |
| 10.0 | 0.997 | 0.007 | 0.994 | 0.014 | 0.992 | 0.012 | 0.985 | 0.014 | 0.972 | 0.025 | 0.960 | 0.029 | 0.948 | 0.032 | 0.941 | 0.033 | 0.935 | 0.032 | 0.930 | 0.031 |
| 11.0 | 0.997 | 0.006 | 0.994 | 0.013 | 0.992 | 0.012 | 0.988 | 0.013 | 0.978 | 0.019 | 0.964 | 0.030 | 0.955 | 0.029 | 0.945 | 0.032 | 0.941 | 0.029 | 0.934 | 0.032 |
| 12.0 | 0.997 | 0.008 | 0.994 | 0.014 | 0.992 | 0.012 | 0.990 | 0.015 | 0.982 | 0.016 | 0.970 | 0.022 | 0.959 | 0.030 | 0.950 | 0.032 | 0.942 | 0.034 | 0.938 | 0.032 |
| 13.0 | 0.997 | 0.006 | 0.995 | 0.009 | 0.992 | 0.014 | 0.990 | 0.016 | 0.983 | 0.017 | 0.975 | 0.020 | 0.964 | 0.023 | 0.953 | 0.029 | 0.946 | 0.031 | 0.941 | 0.029 |
| 14.0 | 0.997 | 0.006 | 0.995 | 0.015 | 0.992 | 0.014 | 0.990 | 0.013 | 0.986 | 0.015 | 0.978 | 0.020 | 0.967 | 0.029 | 0.958 | 0.030 | 0.952 | 0.026 | 0.945 | 0.032 |
| 15.0 | 0.997 | 0.006 | 0.995 | 0.009 | 0.992 | 0.010 | 0.990 | 0.013 | 0.987 | 0.016 | 0.980 | 0.021 | 0.972 | 0.022 | 0.962 | 0.025 | 0.952 | 0.033 | 0.945 | 0.033 |

**References**

57. Whitehead, H. Formations of foraging sperm whales, *Physeter macrocephalus*, off the Galapagos Islands. Can J Zool. 1989;67: 2131-2139.

58. Watkins W, Schevill W. Sperm whale codas. J Acoust Soc Am. 1977;62: 1485-1490.
