# Supplementary material for: Abundance and Distribution of Sperm Whales in the Canary Islands: Can Sperm Whales in the Archipelago Sustain the Current Level of Ship-Strike Mortalities?
Source: PLoS One. 2016 Mar 21;11(3):e0150660. doi: 10.1371/journal.pone.0150660 (PMC4801403; doi:10.1371/journal.pone.0150660)
Supplement: S2 File — (DOCX) [file pone.0150660.s003.docx]

**S2 File. Data imported into Distance program**

**Table A. Formatted data for import into Distance program.** There is a column for each block name, block area, transect name, transect length on effort and perpendicular distance to individual animals. There is a row for each detection.

| **Block name** | **Block area (km^2^)** | **Transect name** | **Transect effort (km)** | **# animals detected** | **Perpendicular distance (m)** |
| --- | --- | --- | --- | --- | --- |
| 1 | 5128.9 | 101 | 55.6 | 0 |  |
|  |  | 102 | 56.2 | 0 |  |
|  |  | 103 | 83.6 | 1 | 1613.4 |
|  |  |  |  | 1 | 2398.7 |
|  |  |  |  | 1 | 1116.7 |
|  |  |  |  | 1 | 659.3 |
|  |  |  |  | 1 | 588.4 |
|  |  | 104 | 9.4 | 0 |  |
| 2 | 1399.7 | 1 | 5.1 | 0 |  |
|  |  | 2 | 26.3 | 0 |  |
|  |  | 3 | 31.8 | 0 |  |
|  |  | 4 | 19.3 | 0 |  |
| 3 | 1758.1 | 5 | 33.1 | 0 |  |
|  |  | 6 | 29.1 | 0 |  |
|  |  | 7 | 16.3 | 0 |  |
| 4 | 1637 | 8 | 19.3 | 0 |  |
|  |  | 9 | 31.6 | 0 |  |
|  |  | 10 | 31.4 | 0 |  |
|  |  | 11 | 26.2 | 0 |  |
| 5 | 2209.7 | 12 | 20.3 | 0 |  |
|  |  | 13 | 36.7 | 0 |  |
|  |  | 14 | 33.2 | 0 |  |
|  |  | 15 | 31.5 | 0 |  |
|  |  | 16 | 21.7 | 0 |  |
| 6 | 1025.9 | 17 | 13 | 1 | 4669.8 |
|  |  |  |  | 1 | 3032.1 |
|  |  |  |  | 1 | 3812.5 |
|  |  |  |  | 1 | 4277.6 |
|  |  |  |  | 1 | 3335.4 |
|  |  |  |  | 1 | 3471.4 |
|  |  |  |  | 1 | 2838.4 |
|  |  |  |  | 1 | 2633.9 |
|  |  |  |  | 1 | 2939.2 |
|  |  |  |  | 1 | 2293.5 |
|  |  | 18 | 37.7 | 1 | 1789.2 |
| 7 | 1360.2 | 19 | 25.8 | 1 | 272.8 |
|  |  |  |  | 1 | 1508.5 |
|  |  |  |  | 1 | 2313.5 |
|  |  |  |  | 1 | 587 |
|  |  |  |  | 1 | 649.4 |
|  |  |  |  | 1 | 649.2 |
|  |  |  |  | 1 | 3940.4 |
|  |  |  |  | 1 | 9531.1 |
|  |  |  |  | 1 | 1155.9 |
|  |  |  |  | 1 | 944.4 |
|  |  |  |  | 1 | 665.9 |
|  |  |  |  | 1 | 2132.8 |
|  |  |  |  | 1 | 2299.2 |
|  |  |  |  | 1 | 5046.2 |
|  |  |  |  | 1 | 6959.2 |
|  |  |  |  | 1 | 1658 |
|  |  |  |  | 1 | 1739 |
|  |  |  |  | 1 | 1193 |
|  |  |  |  | 1 | 1363.6 |
|  |  |  |  | 1 | 568.2 |
|  |  | 20 | 31.7 | 0 |  |
|  |  | 21 | 36.8 | 0 |  |
|  |  | 22 | 21.5 | 0 |  |
| 8 | 935.7 | 23 | 19.8 | 0 |  |
|  |  | 24 | 18.7 | 0 |  |
|  |  | 25 | 8.5 | 0 |  |
| 10 | 3927.2 | 28 | 40.9 | 0 |  |
|  |  | 29 | 38.1 | 1 | 411.4 |
|  |  |  |  | 1 | 417.2 |
|  |  | 30 | 10.4 | 1 | 2223.6 |
|  |  |  |  | 1 | 680.7 |
|  |  | 31 | 20.6 | 0 |  |
|  |  | 32 | 25.4 | 0 |  |
| 11 | 2329.3 | 33 | 28.9 | 0 |  |
|  |  | 34 | 27 | 0 |  |
|  |  | 35 | 26.6 | 1 | 968.6 |
|  |  |  |  | 1 | 4759.5 |
|  |  | 36 | 34.1 | 0 |  |
| 12 | 1206.4 | 37 | 2.8 | 0 |  |
|  |  | 38 | 2 | 0 |  |
|  |  | 39 | 18.4 | 0 |  |
|  |  | 40 | 27.5 | 0 |  |
| 13 | 1194.4 | 41 | 7.3 | 0 |  |
|  |  | 42 | 33.1 | 0 |  |
|  |  | 43 | 22.6 | 1 | 1321.9 |
|  |  |  |  | 1 | 517.3 |
| 14 | 1482.4 | 44 | 21.1 | 0 |  |
|  |  | 45 | 28.1 | 0 |  |
|  |  | 46 | 20.7 | 0 |  |
| 15 | 2954.3 | 47 | 25.8 | 0 |  |
|  |  | 48 | 50.6 | 1 | 2959.1 |
|  |  |  |  | 1 | 4515.8 |
|  |  |  |  | 1 | 2061.8 |
|  |  |  |  | 1 | 10708.9 |
|  |  |  |  | 1 | 3889.5 |
|  |  |  |  | 1 | 2648 |
|  |  |  |  | 1 | 677.9 |
|  |  |  |  | 1 | 2971.5 |
|  |  |  |  | 1 | 1600.8 |
|  |  |  |  | 1 | 2755.3 |
|  |  |  |  | 1 | 3602.2 |
|  |  | 49 | 52.3 | 0 |  |
|  |  | 50 | 37.6 | 0 |  |
| 16 | 1510.8 | 51 | 21.2 | 0 |  |
|  |  | 52 | 34.7 | 1 | 4838.2 |
|  |  |  |  | 1 | 1041.3 |
|  |  |  |  | 1 | 2788.6 |
|  |  |  |  | 1 | 3412.3 |
|  |  | 54 | 11.9 | 0 |  |
| 17 | 1699.3 | 55 | 23.7 | 0 |  |
|  |  | 56 | 44.4 | 0 |  |
|  |  | 57 | 27.8 | 0 |  |
| 18 | 1213.4 | 58 | 10.2 | 0 |  |
|  |  | 59 | 37.3 | 0 |  |
| 19 | 1922.4 | 60 | 10.3 | 0 |  |
|  |  | 61 | 24.3 | 0 |  |
|  |  | 62 | 28.7 | 0 |  |
|  |  | 63 | 29.7 | 0 |  |
|  |  | 64 | 27.8 | 0 |  |
|  |  | 65 | 18.1 | 0 |  |
| 20 | 2676.3 | 66 | 9.4 | 1 | 2988.6 |
|  |  |  |  | 1 | 764 |
|  |  | 67 | 41.4 | 0 |  |
|  |  | 68 | 46.8 | 0 |  |
|  |  | 69 | 50 | 0 |  |
|  |  | 70 | 22.7 | 0 |  |
| 21 | 5120.3 | 71 | 7.6 | 0 |  |
|  |  | 72 | 26.2 | 0 |  |
|  |  | 73 | 25.9 | 0 |  |
|  |  | 74 | 33.1 | 0 |  |
|  |  | 75 | 30.7 | 0 |  |
|  |  | 76 | 37.3 | 1 | 485.19 |
|  |  | 77 | 38.7 | 1 | 1412.6 |
|  |  |  |  | 1 | 2276.3 |
|  |  |  |  | 1 | 2418.3 |
|  |  |  |  | 1 | 2199.9 |
|  |  |  |  | 1 | 1463.4 |
|  |  |  |  | 1 | 3147.5 |
|  |  | 78 | 3.4 | 0 |  |
|  |  | 79 | 38.7 | 0 |  |
|  |  | 80 | 36.8 | 0 |  |
|  |  | 81 | 22.7 | 1 | 7646 |
| 22 | 3393.6 | 82 | 21.6 | 0 |  |
|  |  | 83 | 22.5 | 0 |  |
|  |  | 84 | 37.7 | 0 |  |
|  |  | 85 | 48.2 | 0 |  |
|  |  | 86 | 16.4 | 0 |  |
| 23 | 5110.9 | 87 | 24 | 1 | 1647.1 |
|  |  |  |  | 1 | 3308.1 |
|  |  |  |  | 1 | 856.8 |
|  |  | 88 | 27.4 | 1 | 1313.9 |
|  |  |  |  | 1 | 2934.7 |
|  |  |  |  | 1 | 3696.3 |
|  |  |  |  | 1 | 2679.3 |
|  |  |  |  | 1 | 3112.3 |
|  |  | 89 | 40.8 | 1 | 1596.9 |
|  |  |  |  | 1 | 430.6 |
|  |  |  |  | 1 | 2002.8 |
|  |  |  |  | 1 | 4696.9 |
|  |  |  |  | 1 | 3725.4 |
|  |  |  |  | 1 | 728.2 |
|  |  |  |  | 1 | 2155.1 |
|  |  | 90 | 16 | 0 |  |
|  |  | 91 | 35.7 | 0 |  |
|  |  | 93 | 8.5 | 0 |  |
|  |  | 94 | 6 | 0 |  |
|  |  | 95 | 13 | 0 |  |
|  |  | 96 | 13.6 | 1 | 1963.1 |
| 24 | 1736.8 | 97 | 28.7 | 0 |  |
|  |  | 98 | 23.6 | 0 |  |
| **Total** | **52932.9** |  | **2668** | **85** |  |

**Table B. Overlapping area determination at transect corners for double count correction**. Overlapping areas of acoustic effort were calculated assuming a strip-width of 2 x ESHW. Surveyed areas were considered as overlapping if the respective periods of overlapping effort were within 12 hours of each other. Where surveyed transects met at corners then an automatic calculation of the overlapping area based on the angle of intersection (θ) of the two transects could be made: Area = ESHW² / tan(θ/2), otherwise a manual calculation of the overlapping area was

made in the program Logger.

| **Transect 1 ID**  **(order surveyed)** | **Bearing of surveyed transect** | **Transect 2 ID** | **Bearing of surveyed transect** | **Do surveyed transects meet?** | **Inter-section angle** | **Overlap area (automatic calculation) (km²)** | **Overlap area (manual measurement) (km²)** |
| --- | --- | --- | --- | --- | --- | --- | --- |
| 89 | 81.5 | 88 | 316.5 | N |  |  | 3.5 |
| 88 | 316.5 | 87 | 70.4 | N |  |  | 17.2 |
| 101 | 23.7 | 102-1 | 237.7 | Y | 34.0 | 56.8 |  |
| 104 | 60.1 | 103 | 204.7 | Y | 35.4 | 54.4 |  |
| 71 | 248.6 | 72 | 153.1 | Y | 84.6 | 19.1 |  |
| 72 | 153.1 | 73 | 269.1 | N |  |  | 1.7 |
| 73 | 269.1 | 74 | 146.8 | N |  |  | 23.9 |
| 75 | 271.5 | 76 | 145.2 | N |  |  | 24.8 |
| 78 | 145.0 | 79 | 272.6 | N |  |  | 9.7 |
| 79 | 272.6 | 80 | 145.1 | Y | 52.5 | 35.2 |  |
| 80 | 145.1 | 81 | 264.0 | N |  |  | 13.9 |
| 66 | 273.4 | 67-1 | 152.9 | Y | 59.4 | 30.4 |  |
| 67-2 | 152.8 | 68-1 | 287.0 | N |  |  | 29.8 |
| 68-2 | 287.0 | 69 | 149.3 | N |  |  | 7.5 |
| 61 | 300.7 | 60 | 30.2 | N |  |  | 15.6 |
| 58 | 237.3 | 59 | 302.8 | N |  |  | 0.1 |
| 37 | 336.5 | 36 | 257.2 | N |  |  | 10.4 |
| 36 | 257.2 | 35 | 10.5 | Y | 66.7 | 26.4 |  |
| 24 | 243.9 | 25 | 158.1 | Y | 94.3 | 16.1 |  |
| 25 | 158.1 | 16 | 288.8 | N |  |  | 15.9 |
| 16 | 288.8 | 15 | 32.3 | Y | 76.6 | 22.0 |  |
| 14 | 280.3 | 13 | 35.1 | N |  |  | 23.7 |
| 13 | 35.1 | 12 | 285.3 | Y | 70.2 | 24.7 |  |
| 12 | 285.3 | 18 | 68.8 | N |  |  | 3.0 |
| 18 | 68.8 | 17 | 107.7 | Y | 141.1 | 6.1 |  |
| 17 | 107.7 | 19 | 217.4 | N |  |  | 7.8 |
| 20 | 98.1 | 21 | 221.5 | Y | 56.6 | 32.3 |  |
| 22 | 281.2 | 21 | 41.3 | Y | 59.8 | 30.2 |  |
| 5 | 293.6 | 6 | 211.7 | Y | 98.1 | 15.1 |  |
| 6 | 211.7 | 7 | 283.6 | Y | 108.1 | 12.6 |  |
| 1 | 164.2 | 2 | 64.9 | Y | 80.7 | 20.4 |  |
| 2 | 64.9 | 3 | 178.7 | N |  |  | 6.2 |
| 3 | 178.7 | 4 | 87.4 | N |  |  | 14.1 |
| 4 | 87.4 | 11 | 57.0 | N |  |  | 3.5 |
| 11 | 57.0 | 10 | 323.7 | Y | 86.7 | 18.4 |  |
| 10 | 323.7 | 9 | 70.7 | N |  |  | 6.9 |
| 9 | 70.7 | 8 | 328.0 | N |  |  | 20.6 |
| 8 | 328.0 | 34 | 72.2 | N |  |  | 8.1 |
| 43 | 4.1 | 42 | 286.7 | Y | 102.7 | 13.9 |  |
| 42 | 286.7 | 41 | 357.7 | Y | 109.0 | 12.4 |  |
| 28 | 291.9 | 29 | 194.8 | N |  |  | 0.8 |
| 29 | 194.8 | 30 | 298.1 | N |  |  | 14.6 |
| 46 | 98.5 | 45 | 8.4 | Y | 89.9 | 17.4 |  |
| 51 | 81.4 | 50 | 310.6 | N |  |  | 20.7 |
| 62 | 227.2 | 63 | 110.7 | N |  |  | 12.2 |
| 84 | 179.0 | 85 | 285.6 | Y | 73.4 | 23.3 |  |
| 85 | 285.6 | 86 | 178.4 | Y | 72.8 | 23.6 |  |
| 57 | 202.9 | 54 | 9.7 | N |  |  | 56.9 |
| 54 | 9.7 | 53 | 258.3 | N |  |  | 10.9 |
| 53 | 258.3 | 52 | 9.4 | Y | 68.9 | 25.3 |  |
| 56 | 113.7 | 55 | 21.6 | Y | 87.8 | 18.0 |  |
| Total |  | 51 |  |  |  | 554.2 | 384.2 |

Total area of overlapping survey effort 938 km²

Length of surveyed transect 2668 km

ESHW (Effective strip half-width) 4.168 km

Effective area surveyed (length of transect surveyed x ESHW x 2) 22,239 km²

Overlapping survey effort as fraction of total survey effort 0.04220

Overlapping survey effort as % of total survey effort 4.22 %

Multiplier for *Distance* (1/(1-overlapping fraction) ) 1.044

(no SE was calculated or used)
