# Supplementary material for: Abundance and Distribution of Sperm Whales in the Canary Islands: Can Sperm Whales in the Archipelago Sustain the Current Level of Ship-Strike Mortalities?
Source: PLoS One. 2016 Mar 21;11(3):e0150660. doi: 10.1371/journal.pone.0150660 (PMC4801403; doi:10.1371/journal.pone.0150660)
Supplement: S3 File — (DOCX) [file pone.0150660.s004.docx]

**S3 File. Data imported into simulation model**

**Table A. Echolocating periods derived from Dtag data.** The echolocating periods are grouped in bins and represent the time elapsed from the start to the end of ‘usual’ clicking in foraging dives. It should be noted that the data imported into the model were grouped in finer time bins than shown in the probability histogram.

| **Time (min)** | **Coumts** |
| --- | --- |
| 24.98 | 2 |
| 25.42 | 2 |
| 26.71 | 1 |
| 27.15 | 1 |
| 28.01 | 1 |
| 28.45 | 1 |
| 28.88 | 2 |
| 29.74 | 1 |
| 30.18 | 4 |
| 30.61 | 3 |
| 31.04 | 4 |
| 31.47 | 1 |
| 31.91 | 2 |
| 32.34 | 2 |
| 32.77 | 6 |
| 33.20 | 2 |
| 33.64 | 1 |
| 34.07 | 3 |
| 34.50 | 3 |
| 34.94 | 4 |
| 35.37 | 6 |
| 35.80 | 1 |
| 36.23 | 1 |
| 36.67 | 3 |
| 37.10 | 3 |
| 37.53 | 2 |
| 37.96 | 3 |
| 38.40 | 2 |
| 38.83 | 2 |
| 39.26 | 1 |
| 39.70 | 1 |
| 40.56 | 1 |
| 40.99 | 5 |
| 41.86 | 1 |
| 42.72 | 1 |
| 44.02 | 1 |
| 44.89 | 1 |

**Table B. Not echolocating periods derived from Dtag data.** The not echolocating periods are grouped in bins and are the sum of the silent descent, silent ascent and the time spent at the surface (inter-dive interval) during a dive cycle. It should be noted that the data imported into the model were grouped in finer time bins than shown in the probability histogram.

| **Time (min)** | **Counts** |
| --- | --- |
| 12.236 | 3 |
| 13.165 | 2 |
| 14.094 | 4 |
| 15.022 | 10 |
| 15.951 | 9 |
| 16.88 | 6 |
| 17.808 | 7 |
| 18.737 | 13 |
| 19.666 | 10 |
| 20.595 | 5 |
| 21.523 | 1 |
| 22.452 | 2 |
| 23.381 | 1 |
| 25.238 | 1 |
| 26.167 | 1 |
| 36.383 | 1 |
| 40.098 | 1 |
| 69.817 | 1 |
| 132.97 | 1 |
| 145.04 | 1 |
| 180.34 | 1 |
